# Animals in Iodine Deficiency or Sulfadimethoxine Models of Thyroid Damage Are Differently Affected by the Consumption of Brassica Sprouts

**DOI:** 10.1007/s12011-019-01694-7

**Published:** 2019-03-29

**Authors:** Paweł Paśko, Ewelina Prochownik, Mirosław Krośniak, Małgorzata Tyszka-Czochara, Renata Francik, Monika Marcinkowska, Jakub Sikora, Mateusz Malinowski, Paweł Zagrodzki

**Affiliations:** 1 Department of Food Chemistry and Nutrition, Medical College, Jagiellonian University, Medyczna 9, 30-688 Kraków, Poland; 2 Department of Bioorganic Chemistry, Medical College, Jagiellonian University, Kraków, Poland; 3 Department of Pharmaceutical Chemistry, Medical College, Jagiellonian University, Kraków, Poland; 4 Institute of Agriculture Engineering and Computer Science, Faculty of Production and Power Engineering, University of Agriculture in Krakow, Kraków, Poland

**Keywords:** Iodine deficiency, Brassica sprouts, Rutabaga, Thyroid gland, Immunology

## Abstract

The study was primarily aimed at investigating the effect of brassica sprout consumption, namely rutabaga (*Brassica napus* L. var. *napobrassica*) sprouts (R) generally recognized as antithyroid agent due to its goitrogenic substance content, on hematological, biochemical, and immunological parameters in rats. Sprouts were tested alone and in a combination with other antithyroid factors, such as iodine deficiency (RDI) and sulfadimethoxine (RS). The expression of the heme oxygenase-1 (HO-1) gene in the thyroid as a stress-inducible protein was determined. The thermographic analysis was also estimated. The intake of rutabaga sprouts by healthy rats did not reveal any significant, harmful effect on the thyroid function. Both body temperature and expression of HO-1 remained unchanged in response to the consumed sprouts. In animals with hypothyroidism, rutabaga sprouts enhanced the negative effect of iodine deficiency or sulfadimethoxine ingestion on the organism by increasing the WBC (RDI), TNF-α (RS), creatinine (RS), and triglyceride (RDI and RS) levels, as well as decreasing PLT (RS) level. Moreover, rutabaga sprout consumption by rats with iodine deficiency and sulfadimethoxine decreased their body temperature. Additionally, the concomitant administration of sprouts and iodine depletion significantly reduced the expression of HO-1 in the thyroid. The results may prove useful in confirming rutabaga sprout consumption to be safe, though the seeds of this vegetable provide a well-known antithyroid agent. Our results have shown that rutabaga sprout consumption may be also a factor that enhances the negative clinical features only when combined with iodine deficiency and sulfadimethoxine ingestion.

## Introduction

*Brassicaceae* known also as *Cruciferae* is a plant family that consists of 350 genera, among which the genus *Brassica* is particularly common. Brassica vegetables are an important part of daily diet in European and other countries [1]. However, they contain goitrogenic compounds that cause thyroid hormone synthesis to change, and the structure and size of the gland to vary. Rutabaga sprouts (*Brassica napus* L. var. *napobrassica*) were evaluated as a potential new example of functional food with proapoptotic effects [2]. Rutabaga roots are a potential source of polyphenols, glucosinolates, and vitamin C [2, 3]. As the plant belongs to the *Brassicaceae* family, the vegetable that supposedly has a harmful effect on the thyroid function, it was decided to investigate such an aspect. Our previous work [4] proved that intake of the sprouts by healthy, male animals does not have any harmful effects on their thyroid function. However, in rats with hypothyroidism, the evaluated sprouts enhanced adverse effects of iodine deficiency, or sulfadimethoxine ingestion [4]. Various brassica vegetables are responsible for the damage of the thyroid gland function in rats, pigs, and poultry [5, 6], and a very high consumption of cruciferous vegetables may cause thyroid cancer [7]. Our previous study on broccoli sprouts confirmed that the sprouts did not adversely affect the thyroid function and may have even had a beneficial effect on the thyroid gland antioxidant balance in rats with hypothyroidism [8]. Additionally, progoitrin and glucoerucin, goitrogenic substances isolated from *Brassica napus* L. and from *Eruca sativa* Mill. seeds, respectively, did not cause a significant disturbance in the thyroid hormone profile in the serum of rats [9].

Because the effect of rutabaga sprouts on the thyroid function has been described in details, our next investigation evaluated their effects on selected hematological, biochemical, and immunological parameters of the rat organism. During this study, different models were investigated, such as (1) normal diet, (2) diet with iodine deficiency causing thyroid hyperplasia, and (3) model based on sulfadimethoxine (SDM) added to the animal drinking water as an ingredient (0.025%) and causing thyroid damage by inhibiting the thyroid hormone synthesis. Detailed information about the mechanism of thyroid damage in this models was published previously [4]. The thyroid damage caused by SDM and iodine deficiency was confirmed with serum TSH, thyroid hormones and thyroid histopathology analysis, cytosolic glutathione peroxidase (GPX1), thioredoxin reductase in the thyroid, plasma GPX3 and CAT, and erythrocyte GPX1 [4, 10, 11]; usefulness and applicability in investigation of food influence on the thyroid function were also tested [4].

The present study was conducted to obtain significant information about the sprout influence on the selected hematological, biochemical, and immunological parameters in rats. The expression of the antioxidant-related heme oxygenase-1 (HO-1) gene in the thyroid, and, a thermographic analysis of the animals after an extended experiment were also performed.

## Materials and Methods

### Plant Material

Rutabaga seeds (*Brassica napus* L. var. *napobrassica*) were collected from plants harvested in eastern Poland (Zamość) in 2012. The specimens and the sprouting procedure were described elsewhere [2, 4]. The glucosinolate concentrations in rutabaga 8**-**day sprouts were evaluated with UPLC–MS/MS method, and the following mean values for progoitrin, sinigrin, and glucoerucin were recorded 212.4 ± 12.2 mg/100 g f.w., 154.2 ± 12.3 μg/100 g f.w., and 334.2 ± 81.2 μg/100 g f.w., respectively. Additionally, the concentration of iodine in the rutabaga sprouts was established to reach 6.5 ± 0.6 μg/100 g f.w. [4]. The rutabaga seed sprouting time was based on our previous results, where longer sprouting time was shown to have caused a significantly decrease progoitrin content, the most active goitrogenic compounds of this plant, especially for 10 and 12 days of sprouting, as compared to 8 days [12].

### Animals

**Seventy-two** male (mean weight**,** 123 ± 9 g) 4-week-old Fischer (F344/DuCrI) rats (Charles River, Germany) were kept in plastic cages in an air-conditioned animal room in the Animal House of the Faculty of Pharmacy, Jagiellonian University Medical College. Acclimatization lasted 1 week, then the rats were divided into six groups, each consisting 12 animals, and fed one of the following diets: a standard diet (C), an iodine-deficient diet (DI), a diet with 7% lyophilized rutabaga sprouts (R), an iodine-deficient diet with 7% lyophilized rutabaga sprouts (RDI), a standard diet with 0.025% SDM administered to animals in drinking water (S), or a diet with 7% lyophilized rutabaga sprouts and with 0.025% SDM administered in their drinking water (RS). The detailed procedures, composition of the diet, and the fodder and water intakes were described elsewhere [4]. The animal experiments protocols were approved by the Animal Experimentation Committee of Jagiellonian University, Krakow, Poland (No**.** 105/2012). After 8 weeks, blood was collected from the abdominal aorta under thiopental anesthesia for the biochemical and immunological assays (without hematological parameters evaluated as described below)**,** and the thyroid glands were gathered to evaluate HO-1 expression. Prior to analyses, the samples were stored at − 80 °C.

### Hematological Evaluation

Blood samples (of approximately 60 μL) were obtained from the rat tail veins and placed in plastic Microvette 100 K3E tubes (Sarstedt). A complete blood count was performed using an ABX COBAS MICROS Hematology automated cell counter (ROCHE). The following parameters were determined: red blood cell count (RBC), hemoglobin (Hb), hematocrit (Hct), mean cell volume (MCV), mean cell hemoglobin (MCH), mean corpuscular hemoglobin concentration (MCHC), white blood cell count (WBC), and thrombocyte count (PLT), which were then presented as 10^6^/μL, g/dL, %, fL, pg, g/dL, 10^3^/μL, and 10^3^/μL, respectively.

### Biochemical Analyses

All biochemical analyses of the plasma were performed with kits (Biomerieux, France), according to the manufacturer’s instructions. An ALIZE automatic biochemical analyzer (Lisabio, France) was used in the assays. The following biochemical parameters: glucose (Glu), urea (U), triglyceride (TG), total cholesterol (TC), high-density lipoprotein (HDL), creatinine (Creat), aspartate transaminase (ASPAT), alanine transaminase (ALAT), lactate dehydrogenase (LDH), and alkaline phosphatase (PAL), were evaluated for all the rats in all the groups. The concentration of Glu, U, TG, TC, and HDL was presented as millimolars per liter, and as micromoles per liter for creatinine. The ASPAT, ALAT, LDH, and PAL activities were expressed as units per liter.

### Measurement of Cytokine Levels

Three immunological parameters: interleukin 6 (IL-6), interleukin 10 (IL-10), and tumor necrosis factor α (TNF-α), were evaluated. Rat IL-6, IL-10, and TNF-α ELISA kits were obtained from Diaclone (Besançon, France), and the cytokine determination was performed according to the manufacturer’s instructions [13]. The minimum detectable doses were found to be 19.0, 1.5, and 15 pg/mL, respectively. Cytokine determinations were performed in six rats per group.

### RNA Extraction, Reverse Transcription, and Heme Oxygenase 1 Expression Levels

Total RNA was extracted using a GeneMatrix Universal RNA purification kit (EURx Ltd., Gdańsk, Poland). Reverse polymerase transcription was performed using Reverse Transcription Reagents (Roche, Applied Biosystems, Foster City, CA, USA) and M-MLV Reverse Transcriptase (Promega, Madison, WI, USA) according to the manufacturer’s protocol [14]. Quantitative real-time RT-PCR analyses of the heme oxygenase 1 expression levels in the thyroid glands were performed on ABI PRISM 7300 Sequence Detection System (Applied Biosystems, Foster City, CA, USA) using a commercially available SGqPCR Master Mix (EURx Ltd., Gdańsk, Poland). PCR amplification was performed for 40 cycles. The comparative CT method was used to quantify the relative expression of the genes. The relative quantification of the target gene mRNA expression levels was normalized to the GAPDH mRNA expression.

### Thermographic Investigation

Temperature was recorded with a thermographic camera (ThermaCAM e300) manufactured by FLIR. The procedure was described elsewhere [8].

### Statistical Approach

All of the quantitative data are presented as the mean value ± standard deviation. Between-group comparisons were performed using ANOVA with Dunnett’s post hoc test. Differences with *p* < 0.05 were considered to be statistically significant. A partial least squares analysis (PLS) was applied to reveal the correlation structure between the investigated parameters and to find similarities between the samples. The predictor parameters set consisted of diagnostic biochemical parameters such as glucose, ASPAT, ALAT, TG, TC, HDL, PAL, urea, and creatinine, whereas the blood parameters, namely WBC, RBC, Hb, Hct, MCV, MCH, MCHC, and PLT, were taken as the dependent (response) parameters. PLS model evaluation was performed with the criterion that the percentage of original variation of the predictor parameters explained by the model should exceed 50%, and the corresponding eigenvalues should be higher than 1. The interrelations for parameters were quantified by calculating the correlation weights, i.e., for the considered parameters pairs, the algebraic products of their coordinates and the cosines of the corresponding angles were calculated. The “corresponding angle” was determined by using the two lines connecting the origin of the coordinative system with the points representing both parameters on the final PLS plot. Cluster analysis was conducted in order to detect any similarities between animals when only immunological parameters were taken into account. Statistical analyses were performed using the following packages: SIMCA-P v.9 (Umetrics, Umeå, Sweden; PLS analysis) and Statistica v.12 (Tulsa, OK, USA; PLS diagrams, cluster analysis). The correlation weights were calculated using software delivered by MP System Co. (Chrzanów, Poland). Cluster analysis was performed for the immunological parameters after the standardization of data.

## Results

**Fig. 1:**
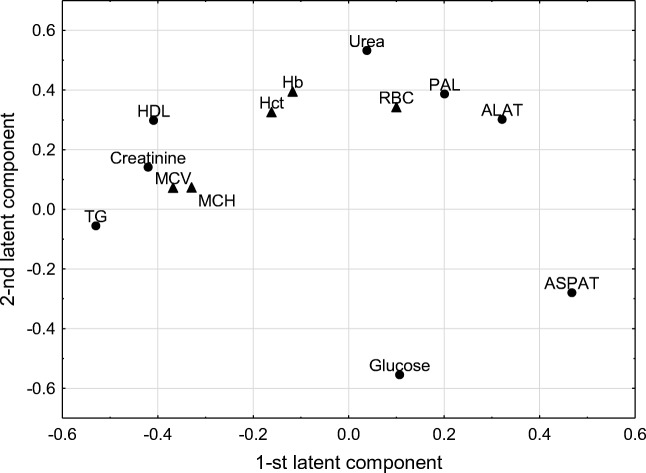
Parameter loadings on the first two latent components in PLS model (dots denote predictor parameters; triangles denote response parameters)

**Fig. 2:**
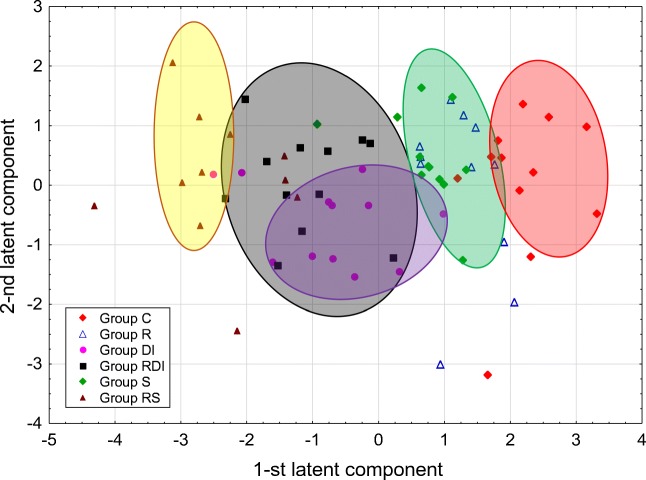
The distribution of the studied samples (individual animals) in the space determined by the first two latent components in PLS model

**Fig. 3:**
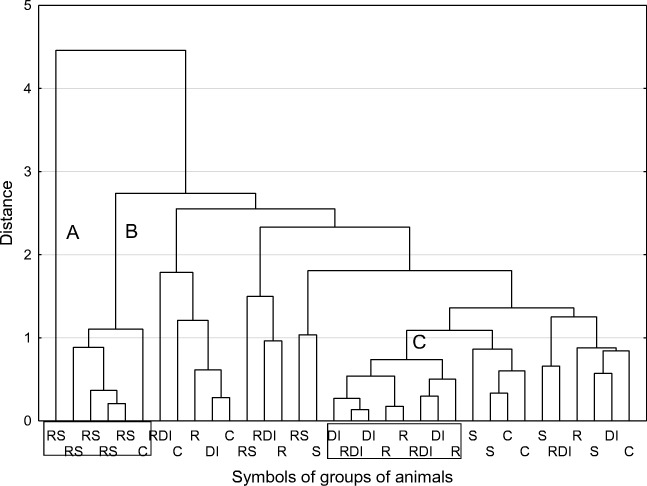
Dendrogram of similarity in immunological parameters in serum among the investigated individuals (method of grouping: average linkage procedure, function of the distance: Euclidean distance; rectangles encompass the individuals belonging to the distinguished clusters A + B and C, respectively; for details see “Discussion”)

**Fig. 4:**
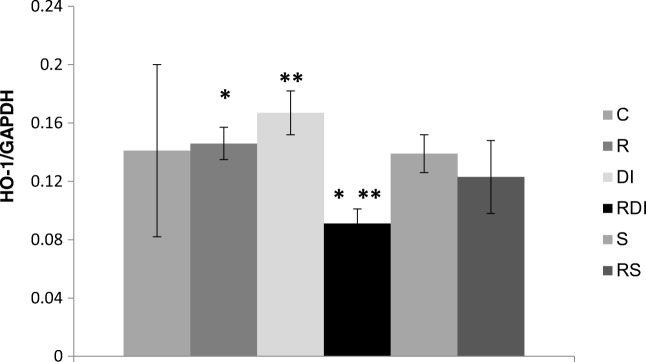
Effect of rutabaga sprouts, deficiency of iodine, and sulfadimethoxine ingestion alone or in combination with other factors on HO-1 expression in the thyroid gland (*n* = 4). Mean values with the same superscript are significantly different between the indicated group at *P < 0.05; **P < 0.01

The results for the hematological, biochemical, and immunological changes and the body temperature are presented in Table 1. The correlation weights based on the PLS model for the hematological and biochemical parameters are shown in Table 2; other results of PLS analysis are shown in Figs. 1 and 2. The cluster analysis for the immunological parameters is shown in Fig. 3. Figure 4 shows the expression of HO-1 in the rats’ thyroid glands.

**Table 1:** Mean values and standard deviations for parameters characterizing blood morphology, biochemical and immunological features and body temperature in all animal groups under investigation

Parameters	C	R	DI	RDI	S	RS	*P* value
*n* = 12	Blood morphology parameters						
RBC (10^6^/μL)	9.6 ± 0.4	9.4 ± 0.2	9.2 ± 0.4	9.6 ± 0.3	9.5 ± 0.4	9.3 ± 0.2	
Hb (g/dL)	14.4 ± 0.5	14.0 ± 0.4	14.0 ± 0.5	14.4 ± 0.5	14.3 ± 0.6	14.7 ± 0.3	
Hct (%)	48.5 ± 3.2	46.4 ± 1.4	47.4 ± 2.5	49.5 ± 1.9	48.5 ± 2.0	50.3 ± 1.4	
MCV (fL)	50.5 ± 1.5	49.5 ± 0.9	51.2 ± 0.8	51.6 ± 0.7	50.8 ± 0.3	54.2 ± 1.0	
MCH (pg/cell)	15.0 ± 0.2	14.9 ± 0.3	15.1 ± 0.2	15.1 ± 0.1	15.0 ± 0.2	15.8 ± 0.3	
MCHC (g/dL)	29.8 ± 1.1	30.1 ± 0.7	29.5 ± 0.8	29.2 ± 0.6	29.5 ± 0.4	29.2 ± 0.6	
WBC (10^3^/μL)	8.3 ± 1.2^a^	7.2 ± 0.8^b^	8.0 ± 1.1^c^	10.5 ± 1.1^abcde^	7.5 ± 1.2^d^	7.0 ± 0.9^e^	abcde***
PLT (10^3^/μL)	563.9 ± 41.8^a^	605.2 ± 43.6^b^	591.0 ± 65.8^c^	603.6 ± 94.2^d^	605.3 ± 28.5^e^	468.9 ± 126.7^abcde^	a*bde***c**
*n* = 12	Biochemical parameters						
Glucose (mmoL/L)	15.13 ± 4.05	15.99 ± 3.40	15.10 ± 2.60	13.30 ± 1.10	13.60 ± 0.61	14.9 ± 2.60	
LDH (U/L)	221.5 ± 140.9	239.4 ± 126.4	206.5 ± 105.9	149.5 ± 41.17	104.2 ± 40.49	168.5 ± 61.27	
Urea (mmoL/L)	4.84 ± 0.23	4.97 ± 0.16	4.70 ± 0.11	4.83 ± 0.30	4.78 ± 0.21	4.77 ± 0.24	
Creatinine (μmol/L)	63.9 ± 6.5^a^	61.3 ± 8.7^b^	71.1 ± 20.5^c^	67.2 ± 13.4^d^	58.7 ± 4.8^e^	81.3 ± 28.3^abcde^	abe***c*d**
ASPAT (U/L)	101.3 ± 15.2^abcd^	85.0 ± 12.5^ef^	69.2 ± 20.1^a^	61.3 ± 14.7^be^	72.2 ± 6.7^cg^	52.1 ± 9.5^dfg^	abcdf***eg*
ALAT (U/L)	73.3 ± 10.5^abcde^	48.9 ± 5.9^afgh^	39.9 ± 7.2^bfi^	41.2 ± 5.9^cj^	59.9 ± 6.4^dgijk^	39.1 ± 6.8^ehk^	abcdeijk***fgh*
TG (mmoL/L)	0.24 ± 0.08^abc^	0.25 ± 0.08^def^	0.74 ± 0.30^adgh^	0.75 ± 0.32^beij^	0.42 ± 0.21^gik^	1.08 ± 0.29^cfhjk^	abcdefk***ghij*
TC (mmoL/L)	1.74 ± 1.03	1.84 ± 0.90	2.30 ± 0.60	1.90 ± 0.50	1.60 ± 0.31	2.01 ± 0.14	
HDL (mmoL/L)	0.78 ± 0.11^ab^	0.81 ± 0.10^cd^	0.96 ± 0.20	1.00 ± 0.17^ac^	0.94 ± 0.12	1.08 ± 0.21^bd^	ad**b***c*
PAL (U/L)	365.9 ± 38.6	345.9 ± 103.4	316.2 ± 63.5	278.0 ± 56.3	369.3 ± 37.3	286.7 ± 26.7	
*n* = 6	Immunological parameters						
TNF-alfa (pg/mL)	39.2 ± 28.0^a^	18.6 ± 2.9^b^	26.4 ± 6.3^c^	33.6 ± 9.9^d^	38.9 ± 7.5^e^	65.8 ± 18.7^abcde^	acde*b***
IL-6 (pg/mL)	63.4 ± 36.1	46.8 ± 26.7	50.2 ± 23.0	48.0 ± 36.0	45.0 ± 12.0	31.0 ± 11.6	
IL-10 (pg/mL)	2.3 ± 0.5	18.5 ± 11.4	2.1 ± 0.2	23.2 ± 19.4	20.2 ± 13.9	40.8 ± 30.3	
*n* = 12	Body temperature						
TEMP (°C)	37.92 ± 0.78^abc^	37.25 ± 1.70	37.88 ± 0.64	36.16 ± 1.22^c^	36.69 ± 0.57^ad^	34.91 ± 0.75^bd^	a* b, c, d***

Mean values with the same superscripts are significantly different between the indicated group at *P < 0.05; **P < 0.01; ***P < 0.001

**Table 2:** Correlation weights for the pairs of correlated parameters (based on PLS model; only correlation weights with absolute values higher than 0.150 are shown)

Pairs of correlated parameters		Correlation weight
Urea	Hb	0.206
TG	Creatinine	0.203
PAL	Urea	0.189
TG	MCV	0.187
Urea	RBC	0.178
TG	MCH	0.165
HDL	Creatinine	0.164
TG	HDL	0.161
Urea	HCT	0.161
Creatinine	MCV	0.153
Glucose	PAL	− 0.168
Glucose	RBC	− 0.169
Glucose	Hb	− 0.195
ASPAT	TG	− 0.225
Glucose	Urea	− 0.286

## Discussion

Our results indicated that neither the addition of rutabaga sprouts to the diet nor the sulfadimethoxine and iodine deficiency exposure caused significant changes in the RBC, Hb, Hct, MCV, MCH, and MCHC parameters. Woyengo et al. [15] reported comparable results: the intake of canola did not adversely affect blood hemoglobin and hematocrit of broilers. In the investigation of similar material, namely broccoli sprouts, significant influence was observed for Hb and MCV [8]. Significant changes caused by rutabaga sprouts were observed for WBC and PLT, but only in groups with thyroid damage caused either by iodine deficiency (increase in WBCs), or by sulfadimethoxine (decrease in PLT). The iodine deficiency and SDM alone did not have any influence on such parameters, which happens to be contrastive to our previous study where DI tended to decrease WBC and PLT, and SDM caused the PLT levels to reduce, although the study was conducted with Wistar rats [8]. Paśko et al. [8] found that in the Wistar rat model’s thyroid damage, most of the significant changes were observed in the iodine-deficient group (a significant increase for Hb, Hct, and MCV; a significant decrease for MCHC, WBC, and PLT) rather than in the group with sulfadimethoxine ingestion (only a significant decrease in WBC). Munters et al. [16] found in a 4-day study that broccoli sprouts caused a significant reduction of lymphocytes and in the monocyte percentage.

A metabolic syndrome in the hypothyroid state includes insulin resistance or glucose intolerance, atherogenic dyslipidemia, endothelial dysfunction, abdominal obesity, proinflammatory state, and thrombosis [17]. Rutabaga sprout consumption, or even thyroid damage models alone, did not cause any significant change in major metabolic state parameters such as glucose, urea, TC, and HDL.

Among the evaluated lipid parameters (TC, TG, and HDL), the maximum changes were observed for TG and HDL. TG increased significantly in the DI group versus the control group, whereas in the group with SDM, it was merely a tendency. Rutabaga sprout consumption alone did not have any effect on the TG concentration, but in a group of rats that received sprouts and SDM, a significant increase was observed. Thyroid damage did not change HDL; however, in the groups of rats with iodine deficiency, or SDM, the addition of sprouts to the diet caused a significant increase in the parameter, which is a beneficial effect. TC remained unaffected; only in the group with DI an increasing tendency was recorded. Cappola and Ladenson [17] indicated that the low thyroid function present increases the atherogenic risk by increasing LDL lipoproteins and TG, and decreasing HDL cholesterol levels, though insulin resistance and obesity may complicate clinical effects of hypothyroidism. Rizos et al. [18] indicated that decreased thyroid function is accompanied by reduced activity of 3-hydroxy-3-methyl-glutaryl-coenzyme A reductase. Additionally, levels of TC and LDL-C are increased in hypothyroidism state which is associated with decreasing of LDL-receptors activity. Furthermore, a decrease in activity of lipoprotein lipase which causes the clearance of TG-rich lipoproteins to decrease and TG level to increase was noted in hypothyroidism, and the raised HDL-C caused by decrease of hepatic lipase activity was also observed [18]. Moreover, increased HDL level may be contributed to the cholesteryl ester transfer protein (CETP) decreased activity, which transfers cholesterol from HDL-C to LDL-C, and to a very low-density lipoprotein (VLDL) as observed in hypothyroidism [19].

Rutabaga sprouts significantly decreased ALAT and had no effect on ASPAT. Thyroid models (DI and SDM) significantly reduced both ALAT and ASPAT. Such results are in contrast to the effect observed in a similar experiment with broccoli sprouts [8], where ALAT was increased in rats fed with broccoli fodder. This effect was contributed to the induction effect of sprouts on the CYP activity, which was supported by Perocco et al. [20], who indicated that glucoraphanin, the most important compound in broccoli, particularly during long-term administration, can induce different isoforms of CYP (CYP1A1/2, CYP3A1/2, and CYP2E1). The increase in the plasma ALAT levels are characteristic for liver injury. In a short-term experiment where sulforaphane was injected intraperitoneally at a dose of 500 μg/kg/days for 3 days, no changes in ASPAT or ALAT were observed [21]. In a long-term experiment, non-significant effect, or any decrease in ASPAT, ALAT, and LDH was observed, which seems a beneficial observation.

No differences were observed in the urea level. The increase in the plasma creatinine level has been used to measure chronic renal failure, and the parameter increased only in the RS group as compared to the other groups. The same effect was observed in a study with broccoli sprouts, where the highest creatinine level was observed in the SDM group [8]. Paśko et al. [8] observed a decrease in the alkaline phosphatase activity in the experiment with rats fed broccoli sprouts, and with the thyroid damage. Further, under the experiment with rutabaga sprouts, the same tendency was found.

A statistically significant PLS model was constructed for the biochemical data and blood parameters. This model explained 53.6% of the original variation of the predictor parameters, and 26.0% of the original variation of the response parameters. The eigenvalues for the first two latent components of this model were equal to 3.07 and 1.22, respectively. WBC, MCHC, PLT, and TC parameters were excluded from the model, as uninformative. Figure 1 and Table 2 show the positive correlation between urea and Hb, PAL, RBC, and Hct, which formed a cluster of mutually positively correlated parameters. Among them, urea, Hb, PAL, and RBC were strongly negatively correlated with glucose. In the second cluster of correlated parameters, creatinine was strongly positively correlated with TG, HDL, and MCV. There was also a negative relationship between ASPAT and TG. The distribution of the examined samples in the space determined by the first two latent components showed apparent differences between group C and all of the other groups; it was particularly visible for RS, RDI, and DI groups, as they were the most distant from group C. The animals from groups S and R, though closer to the controls, were gathered in nearly a separate cluster. Although the DI and RDI groups, and R and S, respectively, overlapped partially, they were still distinguishable in the diagram (see Fig. 2). ASPAT, and the group of parameters containing TG, creatinine, HDL, MCV, MCH were the parameters with the highest positive and negative loadings, respectively, on the first latent component. When comparing Figs. 1 and 2, it is plausible to see that these parameters distinguish animals in the subsequent clusters on the horizontal line. On the other hand, urea and glucose, i.e., the parameters with the highest positive and negative loadings, respectively, on the second latent component, were mainly responsible for slightly different location of groups RDI and DI.

The next part of the experiment was to evaluate the influence of rutabaga sprout consumption, both alone or combined with the thyroid damage factors, on the inflammatory markers. Three cytokines were chosen (see Table 1), two pro-inflammatory (IL-6 and TNF-α) and one anti-inflammatory (IL-10). The rutabaga sprouts introduced to the diet caused an insignificant decrease in IL-6 (R vs. C; RDI vs. DI; RS vs. S), similar to the observation for broccoli sprouts [8]. No significant differences in the IL-10 and TNF-α concentrations were found after sprouts ingested alone, but a beneficial tendency of a decrease in TNF-alpha and an increase in IL-10 was observed. Different models of thyroid damage applied in the experiment resulted in no significantly different effects on the TNF-α, IL-6, and IL-10 levels. The significant observation was recorded for RS group where the two agents, specifically brassica sprout consumption and sulfadimethoxine, were combined. In the RS group, a higher level of TNF-α than that in the control group was observed. Simultaneously, in the same group (RS), the highest level of IL-10 and the lowest of IL-6 were found. This observation was inconsistent with our previous evaluation of the problem [8], and it still escapes explanation. Brassica plants reduce the concentration of proinflammatory and induce anti-inflammatory cytokines [22, 23]. Lin and Li [24] investigated the anti-inflammatory effects of proteinaceous constituents from red cabbage juice and indicated that these compounds showed an anti-inflammatory potential by increasing IL-10, but decreasing the TNF-α secretions using LPS-stimulated mouse splenocytes. Armstrong et al. [25] suggested that IL-10 is a potent inhibitor of the TNF-α release from alveolar macrophages and peripheral blood monocytes. In our model an increase in IL-10 is a supposed part of the mechanism that protects the organism against increasing TNF-α levels. Hypothyroidism is known to be associated with depressed humoral and cell-mediated immunity, and the relationship between cytokines and thyroid disorders, particularly hypothyroidism, might be associated with the progression of thyroid changes [26]. For example, Degertekin et al. [26] suggested that different responses might play a dominant role in a more aggressive, or in the later phase of Hashimoto thyroiditis, rather than in the earlier stages of the disorder. A similar observation was reported by Amadi-Obi et al. [27] for a mouse model of experimental autoimmune uveoretinitis, where the immune response was found to depend on the stage of the disease. Moreover, as our thyroid damage model should induce metabolic changes, the increase of TNF-α is in agreement with the theory where TNF-α may promote atherogenesis through (1) down-regulation of ApoE secretion, which is an important agent in the lipoprotein metabolism; (2) stimulation of vascular cells calcification; and (3) the endothelial dysfunction increase. Additionally, the cytokine can be directly linked to insulin resistance [28], though this aspect needs further exploration.

The cluster analysis, where only immunological parameters were involved, revealed several clusters of animals (Fig. 3). Clusters A and B taken together were the most homogenous and included animals from the RS group, and one individual from group C. As clusters A + B were also most distant from all the others, hence, the modified diet with sulfadimethoxine induced the most pronounced changes to the immunological parameters in rats. In contrast, iodine deficiency, rutabaga supplementation, or a combination of the two treatments caused some individuals to change in a very similar manner, as they formed cluster C containing animals from groups DI, RDI, and from group R. Other animals from different experimental groups were widely distributed in various, non-homogenous clusters. Thus, it was assumed that the experimental conditions did not significantly modify the immunological parameters, with the apparent exception for a model with sulfadimethoxine administered to animals.

HO-1 is a rate-limiting enzyme in the heme catabolism, but its antioxidant, anti-inflammatory, and cytoprotective properties are well-known [29]. By regulating the antioxidant defense pathways, HO-1 may protect cells from environmental stress. However, the overexpression of HO-1 in patients with thyroid cancer can be harmful, as it may enable cancer cells to sustain growth [30]. In our study, in comparison with the control group, none significant influence on the HO-1 expression was exerted either by the iodine deficiency or sulfadimethoxine ingestion (Fig. 4). The addition of rutabaga sprouts to the diet of rats with iodine depletion decreased the gene expression of HO-1, when compared with the group with either a sole iodine depletion, or supplemented only with rutabaga; it follows the changes observed for the thyroid GPX1 activity [4] and may indicate the lower oxidative stress to be caused by the decreased thyroid hormone induction of the reactive oxygen species in the hypothyroid model, whose molecular mechanism remains unknown and such a hypothesis needs to be investigated further. Our data are consistent with the findings of Moon et al. [31], who demonstrated that the phytochemicals of the roots of *Brassica rapa* protected against cisplatin-induced nephrotoxicity by reducing the oxidative stress and was accompanied with the HO-1 protein downregulation. On the other hand, Marzocco et al. [32], who evaluated the biochemical properties of the horseradish root, indicated that this brassica vegetable increased the HO-1 expression; therefore, this aspect needs further detailed investigation. Our analyses revealed that rutabaga sprouts combined with iodine deficiency influence the expression of the HO-1 gene. It is of great interest to study the mechanism that influences the thyroid HO-1 expression in rats with iodine deficiency further.

The results mentioned above are supported by evaluated rat body temperature (Table 1). Neither a diet with rutabaga sprouts nor a diet with iodine deficiency caused significant changes in the rat body temperature to occur. Significant decrease in comparison to the control group were observed for groups S (*p* < 0.05), RS (*p* < 0.001), and RDI (*p* < 0.001). These results should be combined with thyroid hormone concentrations in rats [4] as the lowest levels of fT3 and fT4 were observed for RS group, and this rat group also showed the lowest body temperature (34.91 °C ± 0.75 °C). Thyroid hormones, T3 and T4, can directly activate the thermogenic program in fat cells. T3 is responsible for increasing the metabolic rate via the induction of the transcription of uncoupling protein 1 (UCP1), a major component of the thermogenic program and a specific thermogenic adipocyte marker in the brown adipose tissue. T3 works also to activate the central nervous system to induce thermogenesis [33]. Rutabaga sprouts added to DI diet, or along with SDM, led to greater temperature drops, which indicates that the sprout intake interacts with the iodine deficiency and sulfadimethoxine to lower the thyroid hormones level (data not shown). In similar research, broccoli sprouts did not have any significant influence on the body temperature, though in the group with iodine deficiency–induced thyroid damage and sprouts added to the diet, a significant decrease was found as compared to that in the control group, and that in rats with hypothyroidism [8].

## Conclusions

The intake of rutabaga sprouts by healthy rats did not cause any significant harmful effect on the organ function. The body temperature and expression of HO-1 remained unchanged in response to the sprouts. The results may be useful for confirming the consumption safety for rutabaga sprouts, a vegetable whose seeds provide a known antithyroid agent. In animals with hypothyroidism, rutabaga sprouts enhanced the negative effect of iodine deficiency and sulfadimethoxine ingestion on the organism by either increasing WBC (RDI), TNF-α (RS), creatinine (RS), and triglyceride (RDI and RS), or decreasing PLT (RS) and the body temperature (RDI and RS). Nevertheless, all the aspects, in particular immunological parameters, need a comprehensive evaluation, in terms of the variable amounts of rutabaga sprouts and various experiment duration.
